# “Cyst-ained” research into *Heterodera* parasitism

**DOI:** 10.1371/journal.ppat.1006791

**Published:** 2018-02-01

**Authors:** Parijat S. Juvale, Thomas J. Baum

**Affiliations:** Department of Plant Pathology and Microbiology, Iowa State University, Ames, Iowa, United States of America; THE SAINSBURY LABORATORY, UNITED KINGDOM

Nematodes are roundworms that constitute the phylum Nematoda. Only a small fraction of nematode genera contains plant-parasitic or animal-parasitic species, while the majority of nematodes are free-living [1]. *Heterodera glycines*, the soybean cyst nematode, is a plant-parasitic nematode causing major damage to soybean production worldwide. Annual United States yield loss estimates due to *H*. *glycines* range up to $1.2 billion, likely making this nematode the most serious pathogen threat to sustainable soybean production [2]. While cyst nematode-resistant soybean cultivars are available, they do not control all *H*. *glycines* biotypes present in a given field and, therefore, select for virulent nematode populations that can overcome available resistance genes, leading to a slow but steady erosion of resistance efficacy [3]. Clearly, long-term management of the soybean cyst nematode in modern soybean production will need additional tools, and it is likely that such new tools will be developed from detailed molecular knowledge of the complex *Heterodera* cyst nematode-plant interactions. This short review provides a snapshot of currently unfolding research discoveries from the genus *Heterodera*, which also includes other cyst nematodes, particularly the sugar beet cyst nematode *H*. *schachtii*, which can infect *Arabidopsis* and therefore has been used as a model system. Since nematode effectors (the proteins delivered into host plant tissues to mediate parasitism) are at the forefront of nematode–plant interactions, their identification and functional characterization are heavily emphasized in this manuscript.

**Fig 1:**
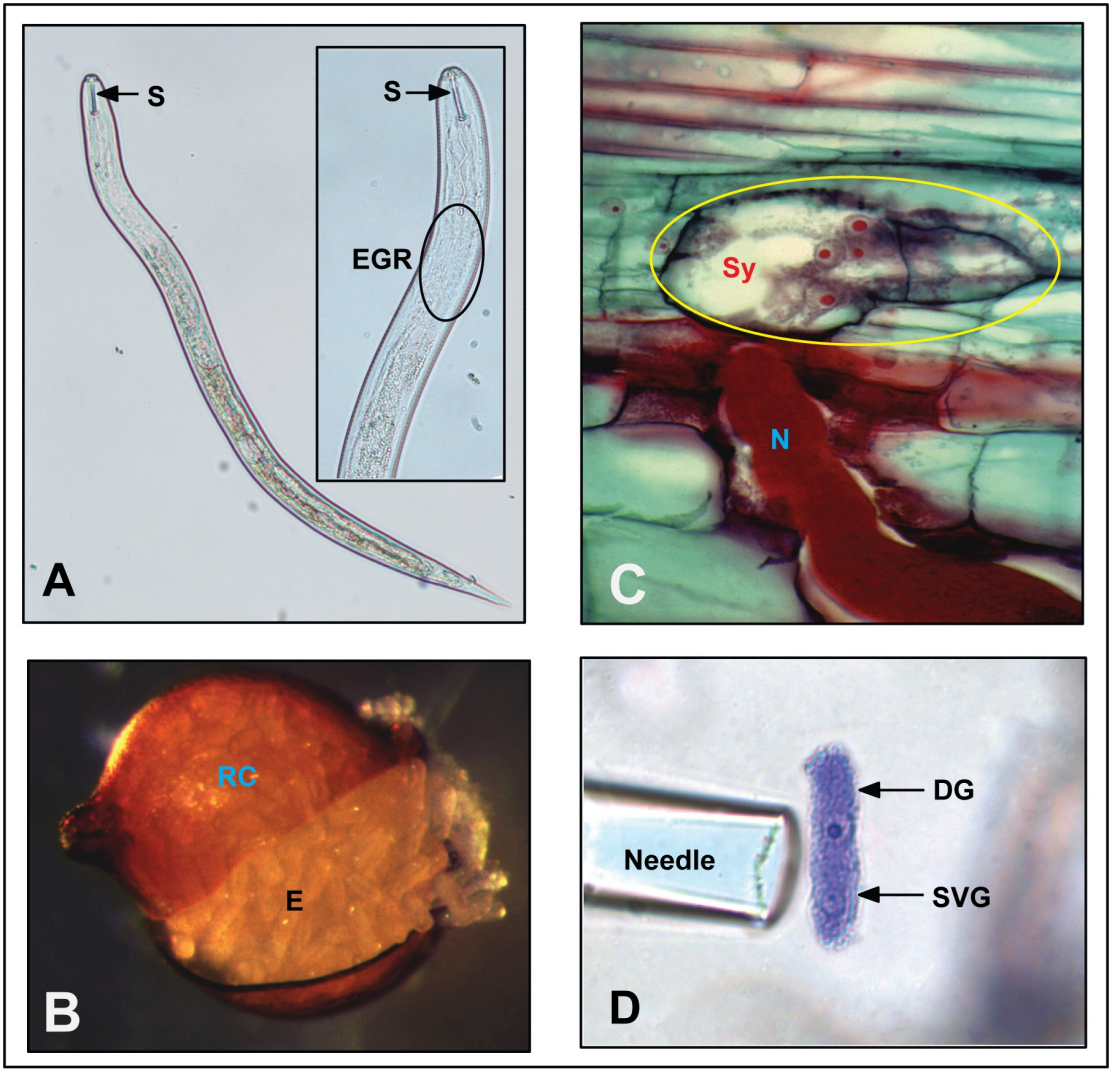
**A**. Infective *Heterodera glycines* juvenile with a close-up of the anterior region showing the stylet (S) and the EGR. **B**. Ruptured RC displaying stored E. *Image by E*. *C*. *McGawley*, *Nemapix*. **C**. Cross section through a SY of a *H*. *glycines* N. *Image by Burton Y*. *Endo*, *Nemapix*. **D**. Microaspiration of isolated and stained DG and SVG gland cells of *H*. *glycines* showing prominently stained nuclei. *Image by Thomas R*. *Maier*, *Iowa State University*. DG, dorsal gland; E, eggs; EGR, esophageal gland region; N, nematode; RC, ruptured *H*. *glycines* cyst; S, stylet; SVG, subventral gland; Sy, syncytium.

*Heterodera* cyst nematodes are soil-borne pathogens. Infective juveniles (Fig 1A) hatch from eggs that are mostly contained in the hardened body wall of the previous generation’s female, which represents the cyst structure giving this nematode group its name (Fig 1B). Infective juveniles migrate toward roots of host plants and penetrate intracellularly into and through the root tissue using mechanical force and cell wall-degrading enzymes delivered through a hollow mouth spear: the stylet (Fig 1A). Interestingly, during their intracellular migration toward the root’s central cylinder, nematodes do not feed but select an initial feeding cell only at the end of their migration, when they become sedentary. At this point, the cyst nematode–plant interactions enter into a new, complex molecular phase of signal exchange, at the end of which the nematode will have reprogrammed a group of several hundred root cells to redifferentiate, partially dissolve their cell walls, and fuse to form a feeding structure: the syncytium (Fig 1C). This new plant organ is the evolutionary advancement that enabled the sedentary parasitic lifestyle of cyst nematodes, as the growing cyst nematodes now require intense levels of nourishment in one single location without the nematodes’ ability to move to new food sources. This also means that a cyst nematode’s survival and reproduction is tightly linked to the proper development and function of the syncytium.

At the heart of syncytium induction and formation are signals sent by the nematode to the initial feeding cell, and the most obvious candidates for such signals are effector proteins produced in three nematode esophagus-associated secretory cells, termed the dorsal and subventral esophageal glands (Fig 1A and 1D), and delivered through the stylet [4]. Identification and characterization of such effectors is thus of prime interest when exploring cyst nematode parasitism.

Microaspiration of esophageal gland-enriched tissues and the construction and sequencing of gland cDNA libraries has identified more than 80 bona fide esophageal gland-produced effector candidates from *H*. *glycines* [5,6]. With the goal of achieving stringent gland cell specificity, the latest efforts to identify additional gland–expressed secretory proteins (i.e., candidate effectors) rely on specifically purifying gland cells (Fig 1D) and using next-generation sequencing technologies [7]. This approach has the potential to unravel the complete effectorome of plant-parasitic nematodes in general and of different strains exhibiting a wide range of virulence phenotypes in particular. Developmental stage- and population-specific effector identification efforts using this approach and bioinformatic prediction pipelines, combined with the development of a comprehensive and well-annotated genome resource (see below), are now unraveling the entire effector repertoire of *H*. *glycines*.

Effector identification has to be followed by functional characterization in order to advance our understanding of parasitism. As in other pathosystems, effectors play critical roles in the suppression of host defenses. In cyst nematode parasitism, however, effectors also are key contributors to reprogramming of plant cells to form syncytia [8–10]. Understanding the mechanisms deployed by *Heterodera* cyst nematodes to suppress or circumvent host defense responses is important, as it will identify vulnerable points for intervention that can aid in devising novel cyst nematode management strategies. Sensing nematode invasion, host plants initiate defense responses ranging all the way to programmed cell death at the infection site [11]. While the mechanisms of triggering defenses are still poorly understood, recent research has revealed that plants are able to detect ascarosides (conserved nematode pheromones) and up-regulate defenses in response to these chemicals [12]. Another mechanistic insight is provided by the recent discovery that *Arabidopsis* receptor kinase NEMATODE-INDUCED LRR-RLK 1 (NILR1) is specifically up-regulated in response to nematode infection and triggers basal immune responses [13]. Naturally, cyst nematodes have evolved the ability to counteract such mechanisms by deploying a suite of effectors. A number of *Heterodera* cyst nematode effector proteins already have been determined to modulate host defense responses. Most of these effectors have been shown to accomplish this feat by physically interacting with and modulating the functions of host proteins involved in different defense response mechanisms.

Cyst nematodes migrate intracellularly through host root tissues causing extensive damage and defense signaling. Delivery of “venom allergen-like protein effectors” (VAPs) during their migratory phase counteracts this defense response. Transgenic Arabidopsis lines expressing a VAP coding sequence showed impaired defense responses to elicitor peptides such as flg22 and were hypersusceptible to various pathogens [14].

One of the first mechanistic insights into defense suppression by *Heterodera* cyst nematodes is the effector-mediated manipulation of polyamine biosynthesis in the host plant through the targeting of plant spermidine synthase. Plants generate reactive oxygen species (ROS) as a response to nematode infection [15]. As a result, syncytia are at risk from the detrimental effects of ROS. Polyamines such as spermidine can effectively scavenge ROS [16,17], thus manipulating the polyamine biosynthetic pathway in the syncytium to generate elevated levels of spermidine could be an effective strategy to protect the syncytium from oxidative stress. *Heterodera* cyst nematodes appear to exploit this mechanism by deploying effector 10A06, which interacts with and activates spermidine synthase, a key enzyme in the polyamine biosynthetic pathway [18].

Elevated expression levels of pathogenesis-related (PR) proteins are primary defense responses observed in host plants under cyst nematode attack [11,19,20]. Growing evidence suggests that cyst nematodes have evolved effectors to counteract PR proteins. For example, Hamamouch et al. [21] described that the elevated expression level of β-1,3-endoglucanase, a PR2 protein family member, is counteracted by *Heterodera* effector 30C02, which physically interacts with this protein. Transgenic plants constitutively expressing a PR2 coding sequence were more resistant, while knock-out mutants were less resistant to the cyst nematodes. On the other hand, plants expressing the effector coding sequence were more susceptible, while plants expressing an RNA interference (RNAi) construct to downregulate effector expression in infecting cyst nematodes were less susceptible.

Another defense-suppressing effector is of interest for a different reason. *H*. *glycines* effector HgGland18 is a strong suppressor of basal as well as hypersensitive cell death immune responses. Bioinformatic and phylogenetic analyses of this effector protein revealed close sequence as well as functional similarity with a nonhomologous effector of the malaria parasite *Plasmodium* that also has immunosuppressive function in its animal host. HgGland18 represents an intriguing example of the convergent evolution of two effectors that arrived at similar structure and function [22].

In addition to effectors with known mechanistic understanding of their function, we also have observed strong defense suppression by a number of *Heterodera* effectors that still await in-depth functional characterization and publication. In fact, most known *H*. *glycines* effectors currently are being assessed for their ability to suppress pattern-triggered and effector-triggered immunity. At the time of writing, we are aware of 11 additional *H*. *glycines* effectors that are able to suppress defenses. It is of particular interest to note that different effector variants, i.e., highly similar proteins within a certain effector group, can exhibit profoundly different activities in such assays. These findings again emphasize the importance of discovering all variants of effectors and detecting correlations among such variants and nematode virulence phenotypes.

As mentioned above, along with robust defense suppression, cyst nematodes also orchestrate massive alterations and reprogramming of host cells at the infection site, which causes these cells to fuse and develop into a syncytium [23]. Not surprisingly, a number of *Heterodera* effectors have been shown to alter cellular structures and developmental pathways in the host plant. Massive modifications in cell wall architecture are essential for the fusion and expansion of root cells at the sites of syncytium formation. *Heterodera* cyst nematodes secrete a suite of cell wall-modifying enzymes such as cellulases and pectinases that can directly modify cell walls. However, their expression profiles show that their main functions lie in the weakening of cell wall barriers during the intracellular nematode migration [24–26]. Instead, cyst nematode infection alters the expression of endogenous cell wall-modifying plant enzymes at the infection sites, which then are responsible for the cell wall modifications during syncytium formation [27]. However, it is likely that cyst nematode effectors have a key role in triggering and modulating the expression changes of these plant genes. In addition, *Heterodera* nematodes also secrete a cellulose-binding protein effector that physically interacts with the host cell wall enzyme pectin methyl esterase and modulates its activity to aid parasitism by influencing cell wall changes [28].

Important functions in host cell modifications observed during cyst nematode infection can also be attributed to phytohormones. These signaling molecules are involved in many facets of plant development, and not surprisingly, they—especially auxin and cytokinin—have also been implicated as key players during syncytium development [19, 29–32]. It should therefore come as no surprise that studies discovered that host proteins involved in auxin functions are targeted by cyst nematode effectors [33, 34]. In addition, there is mounting evidence that *Heterodera* cyst nematodes possess genes essential for cytokinin biosynthesis, which suggests that they have the potential to produce and secrete active phytohormone analogs to induce changes at the infection site [35].

Similar to delivering nematode-produced phytohormones that could alter the endogenous hormone response, *H*. *glycines* secretes effectors with similarity to endogenous plant-signaling peptides. This “ligand mimicry” is best illustrated by the CLE-type effectors of *H*. *glycines*, which dramatically alter soybean cell-to-cell signaling and root development [36–38]. *Heterodera*-produced annexin-like effectors represent another example of mimicry. Cyst nematodes secrete annexin-like effectors to mimic plant annexins, which are involved in a variety of developmental and defense response pathways [39,40]. The constitutive expression of the *Heterodera* annexin-like effector in an annexin knock-out *Arabidopsis* mutant restored the mutant, underscoring this effector’s function [39].

Syncytium development is associated with a massive change in transcript abundances at the infection site [41–44]. Recent studies have begun to describe how *Heterodera* cyst nematodes exploit intrinsic plant mechanisms to control transcription on a global scale. For example, *H*. *glycines* infection results in large-scale methylation changes in the soybean genome [45]. In other words, *H*. *glycines* parasitism triggers significant epigenetic changes in its host, which influences transcript abundances. Furthermore, work in our laboratory produced functional data for an effector that causes DNA methylation changes. Other examples of cyst nematodes hijacking endogenous plant response mechanisms to amplify local nematode stimuli into large-scale systemic responses in the host plant are the reported changes in plant microRNAs during nematode infection [46, 47]. For example, miRNA396 abundance changes dramatically following nematode infection, and miR396 modulations alter the mRNA abundance of approximately half of the *Arabidopsis* genes reported to change in the syncytium [48]. Similarly, expression of miR827and miR858 are altered by cyst nematode infection and serve as amplification mechanisms to cause critical plant gene expression changes [49,50].

Although above-mentioned studies show that *Heterodera* cyst nematodes utilize various strategies and deploy specific effectors to target and modulate cellular pathways, our mechanistic knowledge of cyst nematode parasitism is still at an early stage. Unraveling the entire effector repertoire and functional characterizations are important steps toward developing exhaustive knowledge about cyst nematode parasitism. In this regard, one of the most important resources is a fully sequenced and annotated genome, which is not yet publicly available for any *Heterodera* species at the time of this writing, while high-quality genome assemblies of several *Globodera* cyst nematode species already are available and have significantly benefited researchers [51,52]. A multidisciplinary research team has recently resolved this bottleneck and has produced a high-quality *H*. *glycines* genome assembly that will be released shortly. Already, this genome assembly has allowed comprehensive secretome analyses that promise to expand the currently known effector repertoire considerably. Furthermore, this genome sequence resource is shedding light on the mechanisms of effector evolution and the proliferation of effector variants. Along with the actual annotated *H*. *glycines* genome, work is progressing to create an online web presence that should serve as a comprehensive resource for all *H*. *glycines* genome, transcriptome, effectorome, and proteome data.

While having a high-quality *H*. *glycines* reference genome is critically important, it is only a first step in capturing *H*. *glycines* genomic variability. As a part of the *H*. *glycines* genome project, multiple populations collected from diverse geographical locations or inbred for virulence traits have been and are being sequenced. In short, a boon of novel genomic information is due to be released in the near future, and *H*. *glycines* ‘omics’ research is entering into a new phase of discovery. Such resources will be instrumental in targeting ambitious research goals such as unraveling the genotypic bases of *H*. *glycines* virulence and the potential for molecular diagnoses of *H*. *glycines* strains that can overcome host resistance genes. Collectively, these advances in basic research will enable university and industry researchers to develop and implement novel control mechanisms.

*Heterodera*–host plant interactions are complex, and the recalcitrant nature of *Heterodera* nematodes toward any reverse genetic modifications have long complicated basic research into the molecular mechanisms of cyst nematode parasitism. However, recent advances in conjunction with novel ‘omics’ resources are changing the landscape and give rise to a promising outlook, particularly for *H*. *glycines* research projects with the ultimate goal of resolving this impediment to soybean production. “Cyst-ained,” multipronged, and well-funded research efforts, as described above, are required to develop novel, broad spectrum, and long-lasting resistance resources against this devastating pathogen.
